# The endocrine stress response is linked to one specific locus on chromosome 3 in a mouse model based on extremes in trait anxiety

**DOI:** 10.1186/1471-2164-13-579

**Published:** 2012-10-31

**Authors:** Mariya Gonik, Elisabeth Frank, Melanie S Keßler, Darina Czamara, Mirjam Bunck, Yi-Chun Yen, Benno Pütz, Florian Holsboer, Thomas Bettecken, Rainer Landgraf, Bertram Müller-Myhsok, Chadi Touma, Ludwig Czibere

**Affiliations:** 1 Max Planck Institute of Psychiatry, Munich, Germany; 2 Current address: Institute for Stroke and Dementia Research, Ludwig Maximilian University, Munich, Germany

**Keywords:** F2, Corticosterone, Stress response, HPA axis, QTL

## Abstract

**Background:**

The hypothalamic-pituitary-adrenal (HPA) axis is essential to control physiological stress responses in mammals. Its dysfunction is related to several mental disorders, including anxiety and depression. The aim of this study was to identify genetic loci underlying the endocrine regulation of the HPA axis.

**Method:**

High (HAB) and low (LAB) anxiety-related behaviour mice were established by selective inbreeding of outbred CD-1 mice to model extremes in trait anxiety. Additionally, HAB *vs.* LAB mice exhibit comorbid characteristics including a differential corticosterone response upon stress exposure. We crossbred HAB and LAB lines to create F1 and F2 offspring. To identify the contribution of the endocrine phenotypes to the total phenotypic variance, we examined multiple behavioural paradigms together with corticosterone secretion-based phenotypes in F2 mice by principal component analysis. Further, to pinpoint the genomic loci of the quantitative trait of the HPA axis stress response, we conducted genome-wide multipoint oligogenic linkage analyses based on Bayesian Markov chain Monte Carlo approach as well as parametric linkage in three-generation pedigrees, followed by a two-dimensional scan for epistasis and association analysis in freely segregating F2 mice using 267 single-nucleotide polymorphisms (SNPs), which were identified to consistently differ between HAB and LAB mice as genetic markers.

**Results:**

HPA axis reactivity measurements and behavioural phenotypes were represented by independent principal components and demonstrated no correlation. Based on this finding, we identified one single quantitative trait locus (QTL) on chromosome 3 showing a very strong evidence for linkage (2ln (L-score) > 10, LOD > 23) and significant association (lowest Bonferroni adjusted p < 10^-28^) to the neuroendocrine stress response. The location of the linkage peak was estimated at 42.3 cM (95% confidence interval: 41.3 - 43.3 cM) and was shown to be in epistasis (p-adjusted < 0.004) with the locus at 35.3 cM on the same chromosome. The QTL harbours genes involved in steroid synthesis and cardiovascular effects.

**Conclusion:**

The very prominent effect on stress-induced corticosterone secretion of the genomic locus on chromosome 3 and its involvement in epistasis highlights the critical role of this specific locus in the regulation of the HPA axis.

## Background

To warrant for adequate stress reactions, the hypothalamic-pituitary-adrenal (HPA) axis is activated upon exposure to stressors. As a consequence of such a reaction, corticosterone (CORT) and cortisol, respectively, are secreted in mammals. This is the first phase of the physiological stress response, which already initiates the depletion of further stress hormone secretion via negative feedback mechanisms [1-3]. As demonstrated by various preclinical and clinical studies, exaggerated responses of the HPA axis can lead to continuously elevated stress, considered as one underlying cause in the aetiology of anxiety and depression disorders in humans [3,4]. Anxiety is also reflected at a cognitive level by the individuals' behaviour and strictly connected to physiological responses of central nervous, neuroendocrine and cardiovascular systems [5].

To shed light on the genetic underpinnings of the polygenic, multifactorial trait of anxiety, including its neuroendocrine correlates, multiple approaches have been applied up to date. Genetic engineering has helped to create knock-out and knock-in mice. These models have contributed a considerable amount of knowledge to describe the effects of neurotransmitter and neuromodulator systems, e.g., the corticotropin releasing hormone (CRH), vasopressin (AVP), tachykinin-class neuropeptides or serotonin (5-HT) [6-9]. However, the high number of regulatory mechanisms that are required to maintain the stress response and its return to homeostasis suggests that each molecular player contributes slightly to each phenotype, making it difficult to highlight all the mechanisms involved [10,11].

Compared to genetic engineering, selective breeding strategies have the advantage of keeping the integrity of the genome and simulating the interplay between multiple systems, thus being closer to the clinical situation. In our laboratory, mice of the outbred CD-1 strain were selectively bred for > 20 generations, based on their anxiety-related behaviour on the elevated plus-maze (EPM), resulting in high (HAB) *vs.* low (LAB) anxiety-related behaviour lines. In addition to differences in anxiety-related behaviour, these mice also exhibit comorbid phenotypic differences in other behavioural tests, such as the dark/light box, open field (OF), tail-suspension (TST) or forced swim tests (FST) [12], the latter two being indicative of depression-like behaviour. Hence, using the HAB/LAB mouse model furthered the identification of candidate genes of trait anxiety, including *Avp*, *Enoph1*, *Glo1* and *Tmem132d*[13-16].

We here generated a HAB x LAB F2 intercross and focussed on endocrine system responses as a trait of interest. To reveal the link of genomic loci with the modulation of CORT responses upon stress exposure, we performed linkage analysis followed by an association study to specify the localisation of the QTL. Implementation of multipoint oligogenic segregation and linkage analysis by use of Bayesian Markov chain Monte Carlo (MCMC) method in the presence of missing values increases the power of the study by including the information from a total three-generation pedigree dataset and by sophisticated IBD sharing probability estimations [17]. In the present study, the focus is on the genetic analysis of endocrine phenotypes, while other findings of the linkage of anxiety-related and depression-like behaviour phenotypes (Czibere, unpublished) are largely neglected at this stage. The findings of this study have translational potential for human studies dealing with the dysregulation of the HPA axis, which is regarded as one of the key phenotypes in major depression [4].

## Methods

### Animals

**Figure 1:**
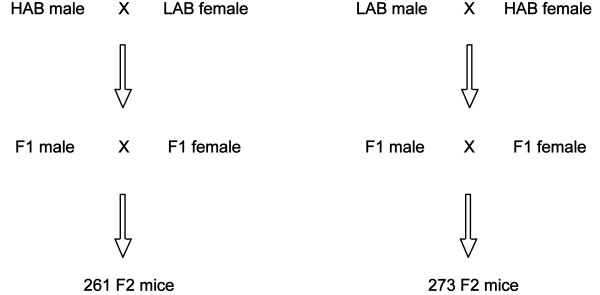
Breeding scheme of the F2 generation from 16 HAB and 16 LAB F0 mice.

All experimental procedures were conducted in accordance with the European Community Council Directive (10/24/1986; 86/609/EEC) and approved by local authorities. All animals tested were bred in the animal facilities of the Max Planck Institute of Psychiatry in Munich as described previously [12-16]. Briefly, mice derived from an outbred mouse strain (CD-1) were bidirectionally inbred, based on their anxiety-related behaviour measured in the EPM test for each generation at an age of seven weeks. After 20 generations of selective breeding, 16 HAB and 16 LAB mice (F0 generation: 8 males and females from each line) were reciprocally cross-mated in order to generate genetically heterozygous animals (F1 generation). F1 animals (N = 58 females and N = 45 males) were further bred with littermates to obtain the F2 generation (N = 534, all males, thereof 273 with a HAB F0 mother and 261 with a LAB F0 mother; Figure 1). In this generation, all differing alleles from the heterozygous F1 parents would segregate freely, thus providing the basis for linkage analysis of the known loci with the behavioural traits. Only male F2 mice were subjected to further analysis to avoid any effects of the oestrous cycle on behavioural and neuroendocrine indices.

Animals were kept under standard housing conditions in groups of two to four per cage (clear plastic, Makrolon type II, 23 × 16 × 14 cm) with a 12-h light/dark cycle (lights on at 07:00 am). Breeding pairs and newborn litters were housed in Makrolon type III (38 × 22 × 15 cm) cages until weaning of the pups at four weeks of age.

### Phenotyping

All behavioural tests were carried out between 08:00 am and 01:00 pm to minimise circadian variations of HPA axis activity. To investigate different traits including anxiety-related behaviour, exploratory drive, locomotor activity, CORT secretion profile and depression-like behaviour, various test paradigms were used [18]. Starting at the age of seven weeks, all animals (F0, F1 and F2) underwent a series of tests including EPM (5 min, 300 Lux on the open arms), TST (6 min), OF (5 min, 60 Lux in the central compartment), elevated platform test (EPF; 5 min), HPA axis reactivity test (HPA-RT; also called stress reactivity test), and FST (6 min, water at 23°C), as already described previously [12,19-21]. Animals were tested once in each paradigm in the order described above with an intertest interval of 48 h. Throughout the test battery, we tried to avoid exposure of animals to two similar tests on two consecutive days. Therefore, as a test for depression-like behaviour, TST was performed 48 h after the first anxiety-related behaviour test (EPM) on day one. To examine long-lasting effects of the relatively stressful TST on the OF test, we exposed 9 CD-1 mice to TST stress two days prior to OF and compared them to 10 unexposed controls. An independent sample of 12 HAB and 11 LAB mice from generation 18 was used to reassure the phenotypic divergence of HAB *vs.* LAB mice in the HPA-RT.

Described just briefly, the procedure for the HPA-RT was as follows. Mice were taken out of their home cages and a first blood sample was taken from their tail veins using glass capillaries. The procedure was limited to 2 min to allow acquisition of basal (i.e., unstressed) values for CORT. The animals were then immobilised for 15 min in 50 ml plastic tubes (with holes at the front and end), and the tubes were covered by a lid to ensure darkness for the restraint stress period. After this 15-min stressor, a second blood sample was taken from a fresh cut less caudally than the first one. Blood samples were processed to obtain at least 10 μl of blood plasma, which was then quantified for CORT using a radioactive immunosorbent assay (DRG Diagnostics, Marburg, Germany) according to the manufacturer’s protocol. All of the samples were assayed in the same batch at the same time. According to the manufacturer’s recommendation, intra-assay variability was reduced to the least possible extent by measuring duplicates. Single measurement values were considered valid, if they were within the range of the standards supplied by the manufacturer.

All behavioural analyses were performed by an experimenter blind to the background of the animals and videotaped for evaluation. Body weight was measured directly after EPM testing. At the age of 14 weeks, animals were single-housed for four days, and their 24-h water consumption was measured as described previously [16]. Then, the animals were decapitated under isoflurane anaesthesia and tail tips were collected for further analysis. Videotapes were scored by an observer blind to the background of the animals using “plus-maze” software for the EPM (E. Fricke, Munich, Germany), Anymaze (Stoelting Co, Wood Dale, IL) for the OF, and Eventlog 1.0 (EMCO software, Reykjavik, Iceland) for TST, EPF and FST, respectively. In the EPM test, an open arm entry is an entry with the forepaws only, corresponding to an entry of > 30% of the full body size, whereas a full open arm entry is an entry with the fore- and hind paws, thus corresponding to > 95% of the full body size.

The results of this study will focus on the phenotypic analyses and on the genetic linkage of CORT-related measurements from the HPA-RT, comprising the CORT concentrations before (basal CORT) and after (stress CORT) the 15-min restraint and the increase in CORT levels (stress CORT minus basal CORT) due to stressor exposure. As the increase in CORT is calculated as mentioned above, it is likely to be correlated, though not identical, with stress CORT, i.e., the two values will not be truly independent.

### Selection of suitable single-nucleotide polymorphisms (SNPs) for HAB *vs.* LAB mice

In an unbiased whole-genome assay, SNPs that consistently differ between HAB and LAB animals (i.e., each line carries the opposite alleles homozygously) were identified, thus allowing for genotyping of F2 mice. To assess a high number of SNPs, the Mouse Medium Density Linkage Panel (Illumina, San Diego, CA) was chosen to determine 1,449 genotypes simultaneously, giving an overview of SNPs available as specific markers for HAB *vs.* LAB animals. Providing the basis for subsequent genotype-phenotype associations in F2 mice, all male and female F0 (N = 16 each) and some F1 mice (N = 18) were genotyped, with the latter serving as controls. For SNPs, where HAB and LAB mice would show the opposite genotype homozygously, F1 mice should exclusively show heterozygous genotypes.

DNA samples were isolated from tail tips using the NucleoSpin Tissue kit (Macherey-Nagel, Düren, Germany). Samples were processed according to the manufacturer’s instructions (Illumina Golden Gate Assay for Sentrix Array Matrix workflow sheets). Fluorescence signals of hybridised samples were captured in the Illumina BeadStation, and genotypes were called from fluorescence intensity clusters using the Illumina BeadStudio software Ver. 3.1.0.0 with the genotyping module Ver. 3.1.12. All intensity clusters were inspected individually and adjusted manually, if necessary. Analysis of genotypes focussed on the detection of valid genotypic SNP markers to distinguish HAB from LAB mice.

### Genotyping of F2 mice

Based on the previously described experiment using the Medium Density Linkage Panel, a custom designed oligo pool (384 SNPs, Golden Gate Assay, Illumina) was set up for genotyping of all 521 F2 mice and, in addition, the 16 HAB and 16 LAB F0 mice, respectively. Therefore, 267 SNPs were chosen from the HAB *vs.* LAB mouse SNP identification experiment and another 116 SNPs were selected from the MGI database to fill up unmapped gaps and to screen selected genes from previously published or unpublished own studies (Additional file 1: Table S1). One SNP (rs3659551) was genotyped twice serving as internal control. Evaluation was performed as described for the Medium Density Linkage Panel. All genomic locations mentioned refer to genome assembly NCBI37/Mm9.

### Statistical analyses

Two groups comparison (Mann-Whitney-*U* test) was conducted for the parental F0 HAB and LAB mice to examine differences in the endocrine stress measures.

A principal component (PC) analysis was applied to examine the phenotypic structure of the F2 mice. PC analysis was used to reveal the phenotypes giving the highest loadings to the total phenotypic variance and to check for correlations between the HPA axis reactivity and the behavioural phenotypes. PCs selected by the stricter broken-stick criterion [22] were kept for analysis. PC analysis was followed by an orthogonal varimax rotation, which resulted in each retained factor bearing a small number of large loadings and a large number of zero (or small) loadings, i.e., the variance of the loadings was maximised. Loadings with absolute values > 0.7 were treated as major loadings. PC analysis and varimax rotation were performed using the open-source statistical software package R ( http://www.r-project.org/). PC analysis was carried out using the normalised phenotypic data of male mice only.

The Loki package [23,24] for multipoint IBD estimations and oligogenic model for segregation and linkage analysis were used to benefit from the entire three-generation pedigree and to implement reversible jump MCMC techniques for likelihood estimations and Bayesian approaches. A quantitative trait was modelled as being genetically controlled by diallelic QTL and affected by the sex covariate. The description by Cox *et al.* served as the basis for conversion of physical to genomic distances on the autosomal chromosomes [25].

The Oligogenic model suggests the presence of multiple putative QTLs. The advantage of the Bayesian strategy, in contrast to other linkage methods, is that the number of putative QTLs is not fixed *a priori*. In contrast, this number was treated as an unknown model parameter. The posterior distribution for the number of underlying QTLs was calculated by using an iterative MCMC sampling technique [26].

The Bayes factor [27], or L-score, was computed to determine linkage as follows. For each sampling iteration (100,000 iterations were used for each analysis), the prior probability of finding a QTL linked to a 1 cM bin was 1 / t, where t was the total map length of the genome (approximately 1400 cM) [28]. If, for a particular iteration, there were n QTLs in the model, the prior probability, *P*, of at least one QTL located in the bin was 1 − (1 – 1 / t) n. The posterior probability, *Q*, is 1 or 0 depending on whether at least one QTL is located in the 1 cM bin. The score for each bin was estimated by averaging *Q* / *P* over an interval of iterations, which is supposed to perform a relatively stable outcome (75,000 – 100,000 iteration interval was used). Genomic regions that have a high probability of containing a QTL were expected to have considerably elevated L-scores compared to surrounding regions. The Bayes factors (L-scores) cannot be quantified in terms of a LOD score, but guidelines for estimating the importance of the Bayes factor are given by Kass and Raftery [27]. Twice the natural logarithm of the Bayes factor is on the same scale, as are the familiar device and likelihood ratio test statistics. 2 log (L-score) scores of a range of 2 to 6 were considered positive signals, 6 to 10 were considered strong, and over 10 very strong [27].

To quantify the linkage signal in terms of more common LOD scores, multipoint parametric linkage analysis of quantitative traits was performed by MQScore_SNP software package [29]. *A priori,* a diallelic autosomal QTL is supposed to be placed near each marker. Parameters of the model are estimated during linkage analysis by maximisation of the LOD score. This score compares the null hypothesis that SNP and QTL segregate independently with the alternative hypothesis, i.e., close linkage between QTL and each marker.

To estimate the location of the identified QTL in terms of common confidence interval and to test for epistatic interactions, QTL mapping in the F2 intercross was performed using the open-source statistical software package R/qtl [30]. Since there is no standard procedure available to determine confidence intervals in multipoint linkage analysis, the single-QTL scan was performed by standard interval mapping (maximum likelihood algorithm). The confidence interval of QTL location was estimated by 95% Bayes credible intervals, which are supposed to depend less on marker density [31]. The QTL-by-QTL epistatic interactions were characterized by the p-values for the LOD scores of corresponding epistatic models. The interactions were accepted to be significant if they did not exceed the adjusted threshold of 0.01. To assess the adjusted p-values, a traditional permutation test (N = 1000) was performed for the genomic region of interest [31].

To localise the most prominent markers more exactly, we additionally implemented the WG-Permer software ( http://www.wg-permer.org) for rapid association analysis on the autosome chromosomes and R ( http://www.r-project.org) for association analysis on X chromosome in male F2 mice. The Bonferroni correction for multiple testing was applied. The level of significance for all association tests was set to 10^-4^. An allelic model was used to make the results comparable with those of the linkage analysis mentioned above [23].

The gene functional classification tool available at the “Database for Annotation, Visualization and Integrated Discovery” (DAVID; http://david.abcc.ncifcrf.gov/) [32,33] was used to retrieve the functional gene categories overrepresented among the genes situated in the region of the identified QTL. Analysis of the enriched functional-related gene groups was carried out with the DAVID-based Fisher's exact test, and results were Bonferroni corrected.

## Results

**Figure 2:**
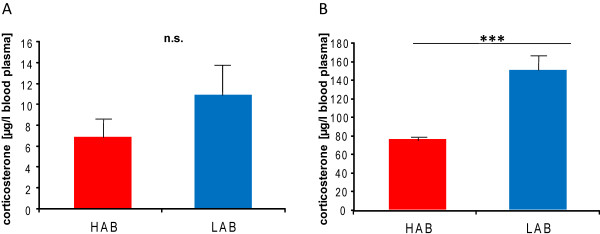
**Corticosterone levels in the blood plasma of male HAB *vs. *LAB mice (A) under basal conditions, (B) after a 15-min restraint stressor.** Data are shown as mean ± standard error of the mean (SEM) with N (HAB) = 12 and N (LAB) = 11; *** p < 0.001.

Phenotypic assessment of the parental F0 HAB and LAB animals (group comparisons) confirmed the previously described data on comorbid anxiety-related and depression-like behaviours [12-15]. In addition, a nominally significant difference was found for the neuroendocrine parameters stress CORT and increase in CORT as measured in the HPA-RT in males (p < 0.05), but not females. As correction for multiple testing abolished this statistic effect (Table 1), the group comparison was replicated in an independent batch of HAB and LAB mice. The significant difference between the two lines could be confirmed (p < 0.001; Figure 2), with LAB mice clearly displaying elevated CORT levels after restraint stress compared to their HAB counterparts. Due to the fact that HABs avoid entering open arms of the EPM and LABs usually spend so much time on the open arm that they rarely enter closed arms, both closed and total arm entries are supposed to be rather unreliable indices of locomotor activity. Accordingly, locomotion is test-dependent (Table 2). Therefore, we rather focus on the total distance travelled in the OF to assess locomotor activity with LAB travelling significantly more than HAB mice. Independent of locomotor activity, the EPF shows nominally significant differences in male mice for explorative behaviour (frequency of head dips; Table 1).

**Table 1:** **Characteristic phenotypes of HAB *vs. *LAB mice (F0 mice, N = 8 per gender and line); values are represented as mean ± standard error of means (SEM); corrected p-values refer to Bonferroni correction and are sorted by p-values of comparison in male mice**

**Phenotype**	**HAB males mean±SEM**	**LAB males mean±SEM**	**p (males)**	**corrected p (males)**	**HAB females mean±SEM**	**LAB females mean±SEM**	**p (females)**	**correctedp (females)**
EPM - percent time on the open arm	4±1	74±2	0.0000	0.0000	6±1	61±6	0.0000	0.0011
EPM - percentage of open arm entries	17±2	63±4	0.0000	0.0000	20±3	53±6	0.0005	0.0115
TST - total immobility time [s]	113±10	4±1	0.0000	0.0002	112±11	7±3	0.0000	0.0010
EPM - closed arm entries	19±1	8±1	0.0000	0.0007	19±1	10±2	0.0020	0.0510
EPM - full open arm entries	0±0	13±2	0.0002	0.0049	0±0	9±1	0.0002	0.0060
EPM - open arm entries	4±1	13±2	0.0016	0.0461	5±1	11±2	0.0102	0.2555
TST - latency to 1st immobility [s]	101±5	232±28	0.0023	0.0633	105±12	235±40	0.0137	0.3423
PHYS - total fluid intake [ml]	6±1	11±1	0.0027	0.0746	NA	NA	NA	NA
OF - total distance travelled [m]	9±2	24±4	0.0042	0.1180	14±2	29±2	0.0003	0.0078
PHYS - daily water intake [ml/g]	0.22±0.03	0.36±0.03	0.0044	0.1231	NA	NA	NA	NA
PHYS - urine osmolality [osmol/kg]	2.9±0.2	2.3±0.2	0.0164	0.4595	NA	NA	NA	NA
EPF - frequency of head dips	4±2	12±3	0.0313	0.8760	4±2	14±6	0.1914	1.0000
**HPA-RT - increase in CORT [μg/l]**	**110±16**	**163±18**	**0.0360**	**1.0000**	**187±27**	**254±26**	**0.0995**	**1.0000**
EPM - latency to 1st open arm entry [s]	87±24	27±5	0.0391	1.0000	118±30	52±12	0.0702	1.0000
EPF - latency to 1st head dip [s]	138±42	33±13	0.0420	1.0000	138±48	110±61	0.7244	1.0000
**HPA-RT - stress CORT [μg/l]**	**118±13**	**165±15**	**0.0499**	**1.0000**	**207±23**	**260±20**	**0.1018**	**1.0000**
EPF - total grooming time [s]	0.84±0.84	4.79±1.77	0.0765	1.0000	1±1	1.25±1.25	0.8789	1.0000
FST - total swimming time [s]	111±25	168±20	0.0994	1.0000	184±27	187±38	0.9448	1.0000
FST - total floating time [s]	165±24	118±21	0.1548	1.0000	128±28	119±34	0.8423	1.0000
OF - total time in the inner zone [s]	7±2	3±2	0.2047	1.0000	6±4	11±4	0.4170	1.0000
EPF - frequency of grooming	0.3±0.3	0.9±0.4	0.2297	1.0000	0.3±03	0.5±0.5	0.6674	1.0000
FST - latency to 1st floating [s]	110±7	95±10	0.2469	1.0000	96±5	121±16	0.1768	1.0000
HPA-RT - basal CORT [μg/l]	6.8±2.5	4.3±0.8	0.3724	1.0000	19±13	11±7	0.6019	1.0000
OF - entries to the inner zone	5.5±2.5	3.1±1.8	0.4510	1.0000	6.8±2.8	8.5±3.5	0.6991	1.0000
EPM - all arm entries	23±2	21±3	0.4732	1.0000	24±2	21±3	0.3112	1.0000
FST - total struggling time [s]	87±9	80±9	0.5799	1.0000	52±7	58±8	0.6015	1.0000
PHYS - body weight [g]	29±1	29±1	0.6776	1.0000	22±1	24±1	0.1141	1.0000
EPM - sum of total arm entries	23±2	21±3	0.4600	1.0000	24±2	21±3	0.5610	1.0000

**Table 2:** Principal component (PC) analysis of behavioural phenotypes in HAB x LAB derived F2 mice sorted by behavioural test paradigms

**Phenotype**	**PC 1**	**PC 2**	**PC 3**	**PC 4**	**PC 5**	**PC 6**	**PC 7**
**HPA-RT – stress CORT [μg/l]**						**−0.97**	
**HPA-RT – increase in CORT [μg/l]**						**−0.96**	
EPM – entries to the middle open arm		−0.96					
EPM – entries to the distal open arm		−0.93					
EPM – time on the distal open arm [s]		−0.92					
EPM – time on the middle open arm [s]		−0.78					
EPM – full open arm entries		−0.77					
EPM – time spent on the proximal open arm [s]					−0.91		
EPM – percentage of open arm entries					−0.89		
EPM – percent time on the open arm					−0.84		
EPM – total time on the open arm [s]					−0.81		
EPM – all open arm entries					−0.77		
FST – total swimming time [s]				0.91			
FST – total floating time [s]				−0.9			
OF – total distance travelled [m]	−0.89						
OF – mean travelling speed [m/s]	−0.88						
OF – total distance in the outer zone [m]	−0.84						
OF – entries to the outer zone	−0.73						
OF – total time in the outer zone [s]			0.97				
OF – total time in the inner zone [s]			−0.96				
OF – total distance in the inner zone [m]			−0.78				
OF – latency to 1st entry of the outer zone [s]			−0.77				
PHYS – daily water intake per g bodyweight [ml/g*d]							−0.95
PHYS – total daily water intake [ml/d]							−0.9

Importantly, even if the preceding test is stressful, it is unlikely to have any impact on behaviour two days later. Indeed, CD-1 mice tested in the TST and CD-1 controls without prior testing showed similar behavioural indices in the OF (Additional file 2: Figure S1).

**Figure 3:**
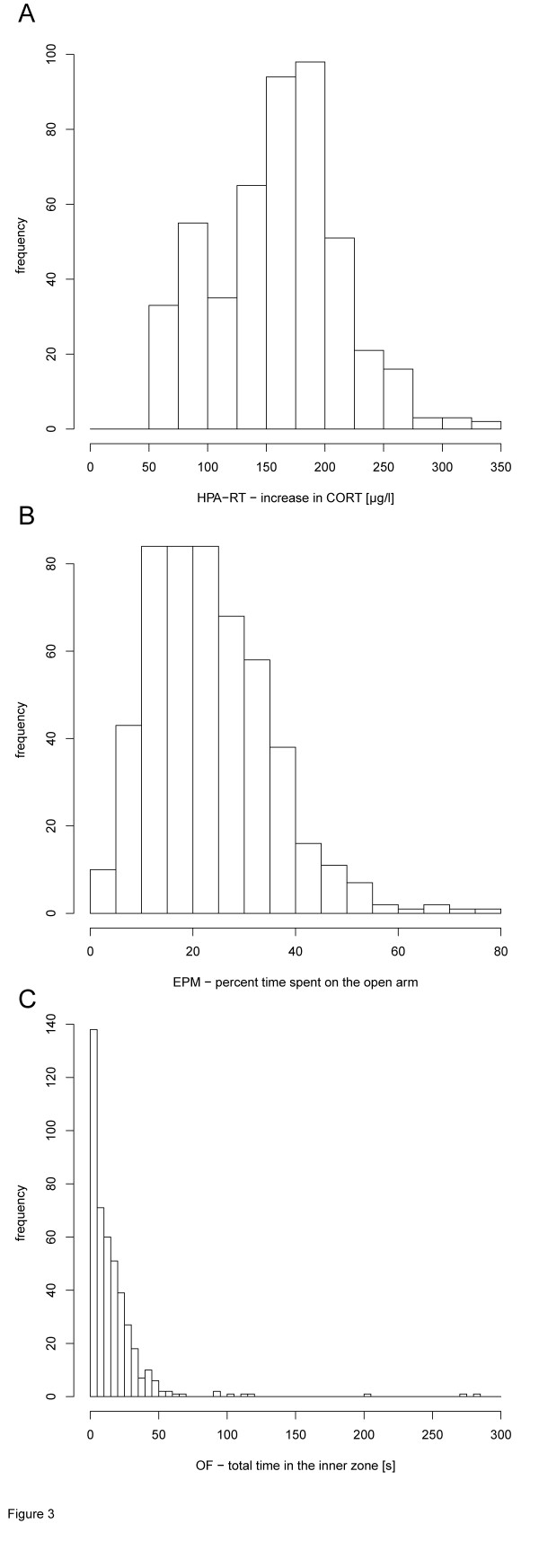
**Histograms of the main phenotypes considered for the F2 mice and the PC analysis.** These included (**A**) the corticosterone (CORT) increase after a 15-min restraint stressor, (**B**) the percent time spent on the open arm of the elevated plus-maze and (**C**) the time spent in the inner zone of the open field.

To structure the phenotypic data obtained from all 44 test parameters in the male F2 mice, PC analysis was performed. Seven PCs were qualified as important and independent according to the selection criterion (Table 2). These 7 PCs accounted for 62% of the total phenotypic variance and included 24 phenotypes providing the highest (i.e., major) loadings of absolute values > 0.7 (Table 2). Basic data distribution is exemplarily shown for the F2 phenotypes HPA-RT (increase in CORT, Figure 3A), EPM (percent time spent on the open arm, Figure 3B) and OF (total time spent in inner zone, Figure 3C).

The comorbidity of anxiety-related and depression-like phenotypes observed in HAB *vs.* LAB mouse comparisons was not reflected by the PC analysis in our F2 animals, leading to high loadings of anxiety-related behaviour as measured in the EPM in PC2, in the OF in PC3 and of depression-like behaviour in PC4 (Table 2). In a similar manner, phenotypes reflecting locomotor activity or exploratory drive in the EPM and OF showed their highest loadings on two separate anxiety-independent PCs (PC5 and PC1, Table 2).

The PC analysis clearly indicated that two of three endocrine parameters, which both reflect the CORT response upon restraint stress (stress CORT and increase in CORT), showed a high correlation (adjusted R^2^ = 0.99). These two endocrine parameters demonstrated the highest loadings (−0.97 and −0.96, respectively) on PC6. Hardly any other PC bore comparably high loadings except for the inner/outer zone time spent in the OF (PC3) and entries to the middle open arm in the EPM (PC1). Thus, PC6, accounting for 4.6% of the total phenotypic variance, is representative of CORT responses to a stressor as measured in the HPA-RT with basal CORT measurements, largely not affecting the overall outcome in F2 mice, leading to similar stress CORT and increase in CORT values. Based on these findings, any of these two endocrine measurements (stress CORT or increase in CORT) might be used as a compound score for the CORT response.

Genotyping of the F2 animals at 384 SNPs (average call rate 93%) resulted in 27 unidentifiable loci, 89 showed no informative value concerning the F2 panel, and one SNP was genotyped twice (showing identical results), leaving 267 SNPs for further testing with informative value for genotype-phenotype associations. From these markers, 233 SNPs were taken from the HAB *vs.* LAB mouse SNP identification experiment and 35 SNPs from those that were additionally screened on the 384 Sentrix Array Matrix (more than 25% of the newly added SNPs were informative for our mouse lines). Eleven markers were only specific for one LAB-subline, thereof two (rs29341895, rs29354500) from the list of SNPs added after the initial screening experiment. These 11 SNPs were not included in the phenotype-genotype linkage due to a shift of allele frequencies, leaving a total of 256 SNPs. This resulted in an average SNP marker density of 1 SNP per 11 Mbp. There were one genomic region of 53 Mbp on chromosome 3 and further five regions between 40 and 45 Mbp in size on chromosomes 2, 4, 10 and 6 for which we did not have any SNP markers. According to their genotypes, all F0 animals could clearly be clustered to their respective HAB or LAB origin.

**Figure 4:**
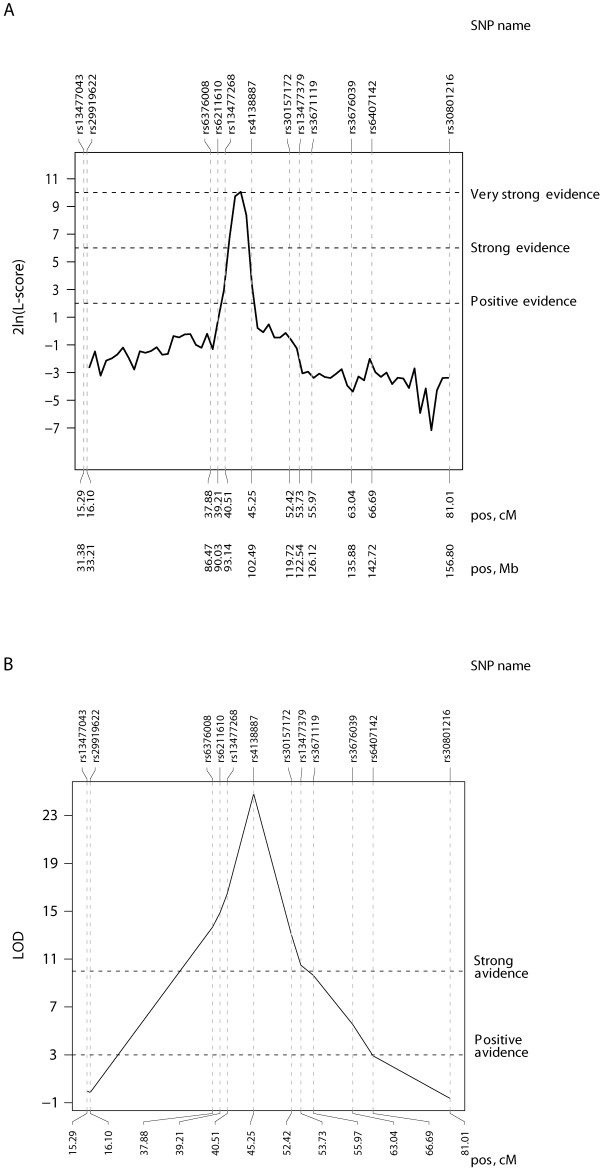
**Linkage of stress corticosterone (CORT) in HAB x LAB-derived three-generation mouse pedigree on chromosome 3.** (**A**) L-score (Bayes factor) multipoint oligogenic linkage analysis of stress CORT using Bayesian Markov chain Monte Carlo approach. (**B**) LOD score two-point parametric linkage analysis of stress CORT.

The stress CORT phenotype was used as a basis for genetic linkage analyses to find the chromosomal regions, which segregate with the trait. First, the MCMC technique was applied genome-wide to take advantage of Bayesian approaches, including a correction for sex in the model. The Bayes factor values (L-scores) were obtained with a step of 1 cM, permitting a better resolution of the QTL for stress CORT. This highlighted a unique region from 40.5 cM (93.1 Mbp) to 45.2 cM (102.4 Mbp) on chromosome 3, showing a very strong positive evidence for linkage (the highest 2ln (L-score) value of > 10 between positions rs13477268 and rs4138887, Figure 4A). Next, a parametric linkage analysis was carried out to estimate the corresponding LOD values on chromosome 3. Stress CORT showed a strong positive linkage signal across chromosome 3 with the highest LOD value of > 23 (Figure 4B).

**Figure 5:**
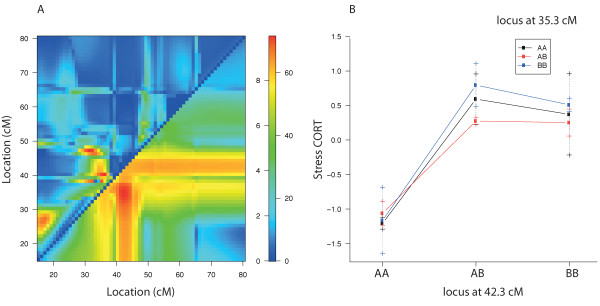
**Two-QTL epistasis on chromosome 3.** (**A**) The upper left triangle contains the LOD scores for the epistasis, e.g., log_10_ likelihood ratio comparing the full model (two QTLs plus interaction) to the additive model (two QTLs); the lower right triangle contains the LOD scores for the full model, e.g., log_10_ likelihood ratio comparing the full model to the null model. (**B**) The phenotype means for each group defined by the genotypes at two epistatic loci.

The maximum likelihood estimate of the QTL location was found at 42.3 cM with the 95% confidence interval indicating the ends of the QTL at 41.3 cM (closest marker: rs13477268 at 40.5 cM) and 43.3 cM (closest marker: rs4138887 at 45.3 cM). The genomic position of the linkage peak (42.3 cM / 97.5 Mbp) is covered by the protein-coding gene *Pde4dip*. The two-QTL scans on chromosome 3 provided evidence for the epistasis (chromosome-wide adjusted p < 0.004), between the identified locus at 42.3 cM and the locus at 35.3 cM (Figure 5).

**Figure 6:**
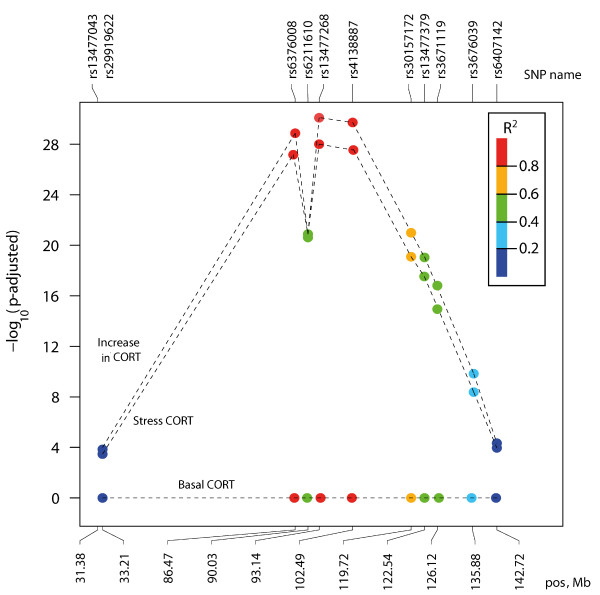
**Association of three phenotypes (stress CORT, increase in CORT, basal CORT) in HAB x LAB-derived F2 mice on chromosome 3.** The correlations R^2^ between the top rs13477268 and surrounding SNPs are coded by a colour bar.

Finally, to check the co-occurrence of traits and markers for CORT-release upon restraint stress, the association analysis of autosomes and the X chromosome was performed for all CORT-related phenotypes: stress CORT, increase in CORT and basal CORT. The significant associations were found for stress CORT and increase in CORT, but not basal CORT. The highest association peaks were placed on chromosome 3 for rs13477268 and rs4138887, followed by rs6376008 with p < 10^-28^ (Figure 6). These 3 SNPs were shown to be highly correlated with R^2^ > 0.8. The pronounced local association minima for stress CORT and increase in CORT (Figure 6) resulted from the shifted allele frequency of the homozygotic genotypes (for rs6376008, rs6211610 and rs13477268). Additionally, besides the strong association signal on chromosome 3, the only other association signals reaching nominal statistical significance (10^-4^ < p < 0.05) were located on the X chromosome, with two pronounced peak regions, the first around 92 Mbp and a second smaller peak around 136 Mbp (Table 3).

**Table 3:** Association analysis of markers on the X chromosome for hypothalamic pituitary adrenal axis activity-related phenotypes (p-values for increase in CORT, stress CORT and basal CORT)

**SNP**	**Position [bp]**	**Gene symbol**	**increase in CORT**	**Stress CORT**	**basal CORT**
rs13483730	39577867	*Stag2*	0.23435	0.28992	0.50204
rs13483765	54787689	*-*	0.04053	0.06132	0.45308
rs13483892	93378516	*-*	0.00094	0.00241	0.21465
rs13483894	93694393	*Heph*	0.00094	0.00241	0.21465
rs6182892	94171734	*-*	0.00094	0.00241	0.21465
rs6221690	127572284	*-*	0.00750	0.01320	0.62939
rs13483997	128648050	*-*	0.01238	0.02112	0.61961
rs13484004	130310074	*-*	0.00564	0.00997	0.69554
rs3697198	132751198	*LOC100040972*	0.00442	0.00802	0.75652
rs13484043	139242663	*Tmem164*	0.00303	0.00525	0.98689
rs13484112	165265740	*Arhgap6*	0.26841	0.37550	0.06247

Importantly, to exclude parent-of-origin effects, calculations made separately for all F2 mice with a HAB F0 mother (N = 273) and with a LAB F0- mother (N = 261) did not show any differences in the effects shown on chromosome 3 (nominal p-value for offspring of HAB F0 mother rs13477268: p < 10^-28^; for offspring of LAB F0 mother: p < 10^-25^).

Combination of linkage and association analyses offered the possibility to approximate the genomic location for the HPA axis response upon restraint stress. Indeed, the linkage study could highlight a single genomic locus, a 5-cM region between markers rs13477268 (40.5 cM / 93.1 Mbp) and rs4138887 (45.2 cM / 102.4 Mbp) on chromosome 3. In addition, the association study revealed the presence of another highly correlated SNP: rs6376008 (37.9 cM / 86.5 Mbp). Therefore, we hypothesised that a set of genes located within that QTL region (37.9 cM - 45.2 cM) might show a well-defined enrichment in specific functional annotation categories. Within the 7-cM QTL, we could identify 391 known mouse genes and conducted a functional classification applying the DAVID analysis tool. Finally, among these 391 genes the analysis revealed a single significantly enriched (Bonferroni corrected p < 0.01) cluster of 5 genes (Table 4). It represents a specific functional class for androgen, oestrogen and progesterone biosynthesis (for linkage to CORT see KEGG enzyme EC 5.3.3.1). Another cluster, including 3 genes and related to cholesterol biosynthesis, was identified (Table 4), but showed only nominal significance (uncorrected p < 0.05).

**Table 4:** Panther functional enrichment analysis of the genomic region strongly linked to the CORT response phenotype

**Enrichment score: 4.76**	**p**	**Fold enrichment**	**Bonferroni corrected p**	**Gene symbol**	**Position, [cM]**	**Position, [Mbp]**
androgen/ oestrogen/ progesterone biosynthesis	8.7E-5	12.3	4.1E-3	*Hsd3b1*	42.89	98.66
				*Hsd3b2*	42.85	98.51
				*Hsd3b3*	42.86	98.55
				*Hsd3b5*	42.82	98.42
				*Hsd3b6*	42.88	98.60
cholesterol biosyntesis	1.4E-2	15.9	4.8E-1	*Hmgcs2*	42.78	98.28
				*Pmvk*	39.18	89.46
				*Fdps*	39.05	89.09

## Discussion

Our results highlight a specific genomic region on chromosome 3 as a major contributor to the variation of CORT response derived from the HAB/LAB mouse model. The strong contribution of genomic factors of this region seems to be essential for regulating stress-related reactivity of the HPA axis.

Here, we described first of all a difference in CORT secretion in HAB *vs*. LAB mice upon a 15-min restraint stressor, with LAB mice secreting more CORT compared to HAB mice. Thus, HAB mice appear to have a rather blunted stress response. Although, in general, high levels of CORT response are described to be associated with high anxiety states [34,35], the opposite is true for HAB *vs.* LAB mice. This finding was replicated in CD-1 mice bred for differences in CORT responses to a stressor, with lower HPA axis reactivity mice showing more passive exploratory behaviour [20]. In addition, a stronger increase in CORT has been demonstrated after social defeat in LAB than in HAB rats, probably due to the more active coping style of the former in response to any kind of stressor [36].

We could not detect any major difference in locomotion of HAB *vs.* LAB mice in the EPM, indicating that the animals’ anxiety-related phenotype is mainly independent of effects based on differing locomotion. Though, we cannot completely reject a confounding influence of locomotion on behaviour, especially as HAB *vs.* LAB male mice exhibited nominally significant differences in locomotion in the OF. On the other hand, we could also detect nominally significant differences in exploratory behaviour on the EPF for HAB *vs.* LAB males, a test much less affected by differences in locomotion.

Linkage studies were applied in rats and mice to a wide variety of complex phenotypes, including alcohol preference, anxiety-related, depression-like and exploratory behaviours or HPA axis function [37-40]. Based on the study by Williams *et al*., where a comparable breeding strategy led to the identification of about 300 genotypic markers useful for complex trait analysis [41], our mouse population seems to fulfil the requirements for linkage analysis. In addition, loading of the phenotypes of different behavioural tests in F2 on independent PCs underlines the good segregation of characteristic traits from the original HAB and LAB mouse lines.

Anxiety phenotypes of EPM and OF tests mainly loaded on two separate PCs showing limited comparability and predictability of measurements from different anxiety tests in freely segregating F2 animals. This is consistent with findings in other studies [42,43] supporting the idea that different paradigms reflect – at least partially – different facets of trait anxiety. Similarly, in the F2 generation, phenotypes representing anxiety, depression and endocrine stress response loaded on separate PCs, reflecting strongly reduced comorbidity of these traits compared to F0 HAB and LAB mice. This resembles findings of another F2 study, where a connection between depression-like behaviour and stress reactivity was rejected [39]. However, as solely based on low loadings of other behavioural phenotypes in the stress-related phenotypic cluster, a connection between these phenotypes cannot be completely denied, being in line with the idea of anxiety and stress reactivity representing multifactorial and polygenic traits with minor contributions of many genes. It is, therefore, likely that associated phenotypes and analogous comorbidities in the clinical situation such as those between anxiety, depression-like behaviours and HPA axis/stress reactivity share contributing genetic factors that are hardly detectable. This is also underlined by a recent study, proposing a network approach to analyse overlapping symptoms in the concept of comorbidity [44].

As stress CORT and increase in CORT are highly correlated (due to the low levels of basal CORT, resulting in almost near-to-identical values for the two parameters) and placed on the independent PC6 representing the only prominent loadings, both might be considered equally representative of the neuroendocrine system response. Stress CORT is exemplarily shown (Figure 4) for genetic analysis. Identified by sequential linkage analysis of trait cosegregation in a full three-generation pedigree and F2 intercross mapping, a CORT-related QTL was detected on mouse chromosome 3 between 41.3 cM (closest marker: rs13477268 at 40.5 cM) and 43.3 cM (closest marker: rs4138887 at 45.3 cM) with a linkage peak at 42.3 cM. Two-QTL scans showed an evidence for epistasis between the region of the peak location at 42.3 cM and the locus at 35.3 cM (closest marker: rs6376008 at 37.9 cM). The association analysis of freely segregating F2 mice derived from a HAB x LAB cross identified a prominent trait co-occurrence with rs13477268 and two highly correlated nearby markers, rs4138887 and rs6376008. The high correlation of nearby SNPs (rs13477268, rs4138887 and rs6376008) points to the presence of a broad linkage disequilibrium (LD) block, indicating that the identified group of significant markers represents the effect of the true locus underlying the neuroendocrine stress response.

Taken together, our findings highlight the region from 37.9 cM to 45.2 cM of mouse chromosome 3 as an endocrine stress response-specific locus. The additionally identified regions on the X chromosome, only showing nominally significant association with the traits stress CORT and increase in CORT, provide an impetus for the involvement of sex-specific mechanisms in influencing the respective trait, which is supported by other studies [45].

Comparative genome analyses of mouse chromosome 3 showed rat and human homologues on chromosomes 2 (rat) and 1 and 4 (human), respectively [46]. The markers that were linked to stress CORT and increase in CORT are not located near to any neurotransmitter receptor gene or other candidate genes for HPA axis regulation mapped to date in mice (according to NCBI, Ensembl and UCSC databases).

A particularly interesting candidate gene for the trait of neuroendocrine stress response is *Pde4dip*, a locus characterised by the maximum likelihood estimate of QTL location and involved in epistasis. The encoded protein, myomegalin, is responsible for cardiac contractility during adrenergic stimulation [47]. The rat homologues of our identified QTL (Figure 4A) are highly overlapping with previously identified QTLs for cardiovascular disease-related phenotypes. These overlaps include rat QTLs for blood pressure and heart rate [48] and bradycardia [49]. As these rat QTLs also include the genomic locus of *Pde5a*, which is capable of modulating acute and chronic cardiac stress responses [50,51], represented by rs13477379 in our study, it makes this gene an interesting additional candidate, although not included in the region of strongest linkage. Particularly as acute CORT responses show a pronounced effect on the cardiovascular system [52], the previously described cardiovascular QTLs in this genomic region are in line with our findings.

The identified QTL is located in a region that also overlaps with a QTL for ethanol-induced CORT responses in mice [53] and is homologous to a QTL for fasting cortisol levels in humans [54]. Both ethanol administration and fasting have been shown to provoke an increase in CORT secretion and glucocorticoid production, similar to acute stress responses [55,56]. Finally, the 7-cM region, identified to be linked to stress CORT in the present study, is completely homologous with one of the QTLs (*Srcrt-1*) Solberg *et al.* described to influence stress CORT in their rat model [40], with the difference that the linkage in our study can be limited to a single, smaller region, which is strongly linked to the stress CORT phenotype. In case of Solberg *et al*., *Srcrt-1* shows the second strongest effect on stress CORT, behind a marker on rat chromosome 6 (homologous to mouse chromosome 12) of five linked genomic loci altogether [40].

The strongly linked 7-cM genomic region on chromosome 3 harbours at least five genes involved in steroid, most prominently glucocorticoid, synthesis (Table 4) [57], emphasising a strong effect on the stress CORT phenotype. Although these genes are primarily focussed on androgen, oestrogen and progesterone synthesis (Table 4), their enzyme products are known to play a vital role in the synthesis of all glucocorticoids, among them CORT (KEGG pathway mmu00140; http://www.genome.jp/kegg/). This strongly supports a conserved genomic and, thereby, heritable effect on stress reactivity. Interestingly, a recent study indicates that corticosteroids can be synthesised endogenously in the brain [58]. Therefore, the genomic region identified in our study might also affect corticosteroids directly in the brain. The second functionally enriched group (Table 4), albeit showing only nominal significance, is related to cholesterol biosynthesis, which is the first metabolite required for glucocorticoid synthesis [59]. Another genomic site containing genes coding for glutamate receptors and other transmembrane proteins (NCBI) is related to the locus at 35.3 cM, which is in epistatic interaction with the identified locus at 42.3 cM.

While many mechanisms of genomic and non-genomic regulation remain largely unclear in terms of activation *vs.* inhibition of the HPA axis [45], our results strongly point to a genetic influence of the 7-cM region. The additionally identified regions on the X chromosome, only showing nominally significant linkage to the stress CORT trait, provide an impetus for the involvement of sex-specific mechanisms in influencing the respective trait, which is supported by other studies [60].

## Conclusion

Taken together, based on a HAB x LAB mouse intercross panel, we have identified one major QTL on chromosome 3 linked to both stress-induced CORT level and the level of increase in CORT, providing a strong basis for the heritability of neuroendocrine stress reactivity and emphasising a critical role of the genes relevant for steroid synthesis. In addition to the neuroendocrine effect, *Pde4dip* covering the position of the linkage peak and *Pde5a* placed in a nearby region overlapping with known cardiovascular QTLs, suggest a comorbid cardiovascular effect driven by the identified locus. Further supported by epistatic effects, our findings highlight a novel aspect towards the understanding of the well-orchestrated mechanisms of stress response regulation. They will contribute to further facilitate the search for candidate genes that are important for the regulation of neuroendocrine systems under stress conditions and, simultaneously, provide deeper insights into their phenotypic outcome, behaviour.

## Competing interests

The authors declare that they have no competing interests.

## Authors' contributions

MG planned and carried out the statistical analyses, EF, MSK and RL conceptualised and carried out the F2 breeding, behavioural assessment and analysis of behaviour, DC participated in the statistical analyses, MB and YCY carried out the behavioural assessment and analysis, BP participated in conceptualising the statistical analysis, FH contributed to shaping the project, TB contributed to planning and carrying out the genotyping, RL conceptualised and supervised the project, BM conceptualised and supervised the statistical assessment, CT participated in planning and carrying out the phenotyping and provided additional neuroendocrine data, LC carried out the behavioural assessment, conceptualised and carried out the genotyping and wrote the manuscript together with MG and RL. All authors have read and approved the final manuscript.

## Supplementary Material

Additional file 1 ** Table S1.** Single-nucleotide polymorphisms (SNP) tested using the custom designed oligo pool (Illumina). Custom designed SNP pool for Illumina Golden Gate Assays to genotype the F2 mice for the current study. Source (1) refers to SNPs, chosen from the Mouse Medium Density Linkage Panel, (2) additional SNPs selected from the MGI database based on genes known from previously published or unpublished studies. Gene association is assumed, if a SNP is located 10 kbp around a gene locus. Click here for file

Additional file 2 ** Figure S1.** Effect of prior tail-suspension test exposure on the behaviour in the open field test. Key parameters assessed in the open field test showed no significant difference in CD-1 mice between control (N = 10, only exposed to the open field test) and tst (N = 9, exposed to the tail-suspension test 48 h before the open field test) mice. Data are shown as means +SEM. Click here for file
