# Supplementary material for: The endocrine stress response is linked to one specific locus on chromosome 3 in a mouse model based on extremes in trait anxiety
Source: BMC Genomics. 2012 Oct 31;13:579. doi: 10.1186/1471-2164-13-579 (PMC3557225; doi:10.1186/1471-2164-13-579)
Supplement: Additional file 1 — Table S1. Single-nucleotide polymorphisms (SNP) tested using the custom designed oligo pool (Illumina). Custom designed SNP pool for Illumina Golden Gate Assays to genotype the F2 mice for the current study. Source (1) refers to SNPs, chosen from the Mouse Medium Density Linkage Panel, (2) additional SNPs selected from the MGI database based on genes known from previously published or unpublished studies. Gene association is assumed, if a SNP is located 10 kbp around a gene locus. [file 1471-2164-13-579-S1.doc]

**Supplementary table 1:** Single-nucleotide polymorphisms (SNP) tested using the custom designed oligo pool (Illumina). Source (1) refers to SNPs, chosen from the Mouse Medium Density Linkage Panel, (2) additional SNPs selected from the MGI database based on genes known from previously published or unpublished studies. Gene association is assumed, if a SNP is located 10kbp around a gene locus.

| **Source** | **SNP identifier** | **Chr.** | **Physical map [bp]** | **Associated gene** |
| --- | --- | --- | --- | --- |
| 1 | mCV23695025 | 1 | 22,398,088 | *-* |
| 1 | mCV24784983 | 1 | 25,476,000 | *Bai3* |
| 1 | rs3677683 | 1 | 27,321,156 | *-* |
| 1 | rs4137502 | 1 | 30,887,618 | *Phf3* |
| 1 | rs3707642 | 1 | 32,568,345 | *Khdrbs2* |
| 1 | rs3683997 | 1 | 35,935,818 | *-* |
| 1 | rs13475827 | 1 | 40,990,938 | *-* |
| 1 | CEL-1_44668113 | 1 | 44,620,787 | *-* |
| 1 | rs13475881 | 1 | 58,439,402 | *-* |
| 1 | rs13475919 | 1 | 73,020,555 | *-* |
| 2 | rs30238170 | 1 | 82,719,300 | *-* |
| 2 | rs30238169 | 1 | 82,720,355 | *5230400G24Rik* |
| 2 | rs30238168 | 1 | 82,720,980 | *5230400G24Rik* |
| 2 | rs30237262 | 1 | 82,722,282 | *5230400G24Rik* |
| 2 | rs30236408 | 1 | 82,726,579 | *5230400G24Rik* |
| 2 | rs30242174 | 1 | 82,749,582 | *-* |
| 1 | UT_1_89.100476 | 1 | 87,014,950 | *Chrnd* |
| 1 | rs13476012 | 1 | 101,,801,487 | *Cntnap5b* |
| 1 | CEL-1_103251925 | 1 | 103,228,922 | *-* |
| 1 | rs3685919 | 1 | 111,528,321 | *-* |
| 1 | rs13476050 | 1 | 112,508,292 | *-* |
| 1 | rs3699561 | 1 | 132,988,758 | *Mapkapk2* |
| 1 | rs3672697 | 1 | 147,028,489 | *-* |
| 1 | rs13476163 | 1 | 148,717,645 | *B830045N13Rik* |
| 1 | rs6393307 | 1 | 152,872,095 | *-* |
| 1 | rs13476187 | 1 | 156,052,564 | *ENSMUSG00000066797* |
| 1 | rs6157620 | 1 | 185,385,733 | *-* |
| 1 | rs3667164 | 1 | 190,511,531 | *Ush2a* |
| 1 | rs6240512 | 2 | 10,900,065 | *100040690* |
| 1 | CZECH-2_15618849 | 2 | 15,594,129 | *-* |
| 1 | rs13476366 | 2 | 19,320,592 | *-* |
| 1 | rs13476503 | 2 | 53,042,643 | *Prpf40a* |
| 1 | rs4223189 | 2 | 61,638,140 | *Psmd14* |
| 1 | rs3664661 | 2 | 71,436,620 | *-* |
| 1 | CEL-2_73370728 | 2 | 73,174,311 | *-* |
| 1 | rs13476639 | 2 | 92,666,968 | *-* |
| 1 | rs6406705 | 2 | 100,200,136 | *-* |
| 1 | rs13476666 | 2 | 101,163,197 | *-* |
| 1 | rs13476689 | 2 | 107,305,294 | *-* |
| 1 | rs13476723 | 2 | 117,118,533 | *Rasgrp1* |
| 2 | chlcdelavp2 | 2 | 130,273,975 | *Avp* |
| 2 | cmlcsnpavp1 | 2 | 130,276,153 | *Avp* |
| 1 | rs13476783 | 2 | 133,686,867 | *-* |
| 1 | rs3664408 | 2 | 161,205,958 | *-* |
| 1 | CEL-2_168586738 | 2 | 168,032,354 | *Nfatc2* |
| 2 | rs31438972 | 3 | 19,586,067 | *Trim55* |
| 2 | rs31145247 | 3 | 19,586,366 | *Trim55* |
| 2 | rs30796162 | 3 | 19,597,491 | *-* |
| 2 | rs31286319 | 3 | 19,598,902 | *-* |
| 1 | rs13477043 | 3 | 31,379,500 | *-* |
| 1 | gnf03.030.222 | 3 | 32,722,005 | *-* |
| 1 | rs6376008 | 3 | 86,465,169 | *Lrba* |
| 1 | rs6211610 | 3 | 90,025,782 | *Rab13* |
| 1 | rs13477268 | 3 | 93,138,727 | *-* |
| 1 | rs4138887 | 3 | 102,493,088 | *-* |
| 1 | CEL-3_120379605 | 3 | 118,794,515 | *-* |
| 1 | rs13477379 | 3 | 122,540,626 | *Pde5a* |
| 1 | rs3671119 | 3 | 126,116,580 | *Arsj* |
| 2 | rs13477411 | 3 | 131,922,067 | *-* |
| 2 | rs6166189 | 3 | 132,079,726 | *-* |
| 2 | rs31556559 | 3 | 132,298,496 | *EG433653* |
| 2 | rs30263909 | 3 | 132,299,981 | *EG433653* |
| 1 | rs3676039 | 3 | 135,880,574 | *Bank1* |
| 1 | rs6407142 | 3 | 142,720,843 | *-* |
| 1 | gnf03.160.599 | 3 | 156,149,851 | *Negr1* |
| 2 | rs3022975 | 4 | 8,073,046 | *Car8* |
| 1 | CEL-4_30653207 | 4 | 30,606,147 | *-* |
| 2 | rs3090720 | 4 | 57,221,105 | *Ptpn3* |
| 1 | rs3708471 | 4 | 76,516,632 | *Ptprd* |
| 1 | rs13477873 | 4 | 101,102,850 | *Ak3l1* |
| 2 | rs3022996 | 4 | 111,044,395 | *4931433A01Rik* |
| 1 | rs3023025 | 4 | 142,772,319 | *Prdm2* |
| 1 | rs13478110 | 5 | 9,741,228 | *-* |
| 1 | rs3714258 | 5 | 12,371,157 | *-* |
| 1 | rs6341620 | 5 | 37,492,799 | *Jakmip1* |
| 1 | CEL-5_45872918 | 5 | 46,008,170 | *-* |
| 1 | rs3664008 | 5 | 54,048,319 | *Rbpj* |
| 1 | mCV23386455 | 5 | 62,987,260 | *Tbcd1d* |
| 1 | rs3667334 | 5 | 83,471,068 | *-* |
| 1 | rs13459087 | 5 | 87,521,105 | *Ugt2b36* |
| 1 | CEL-5_87173557 | 5 | 88,825,844 | *Amtn* |
| 1 | gnf05.084.686 | 5 | 89,982,732 | *Npffr2* |
| 1 | rs3673049 | 5 | 90,116,719 | *Adamts3* |
| 2 | rs31780700 | 5 | 92,889,948 | *Scarb2* |
| 2 | rs29583970 | 5 | 92,983,999 | *-* |
| 2 | rs31786987 | 5 | 93,102,064 | *4932413O14Rik* |
| 1 | rs3661241 | 5 | 98,266,375 | *-* |
| 2 | rs13460000 | 5 | 100,488,240 | *Enoph1* |
| 2 | rs13460001 | 5 | 100,490,027 | *Enoph1* |
| 1 | rs13478433 | 5 | 104,357,982 | *-* |
| 1 | rs13459186 | 5 | 110,534,259 | *Gtpbp6 / Plcxd1* |
| 1 | rs13478483 | 5 | 118,405,617 | *Nos1* |
| 1 | rs13478518 | 5 | 128,264,975 | *Tmem132d* |
| 2 | rs33711358 | 5 | 128,515,999 | *Tmem132d* |
| 2 | rs13478520 | 5 | 128,616,797 | *Tmem132d* |
| 1 | rs6298689 | 5 | 140,240,607 | *Ints1* |
| 2 | rs36247439 | 5 | 149,857,285 | *Hmgb1* |
| 2 | rs29781244 | 5 | 149,862,877 | *Hmgb1* |
| 2 | rs33343556 | 5 | 149,865,874 | *-* |
| 2 | rs36309698 | 5 | 149,868,499 | *-* |
| 2 | rs37452785 | 5 | 149,868,789 | *-* |
| 2 | rs30116240 | 6 | 7,499,262 | *-* |
| 2 | rs30221186 | 6 | 7,503,288 | *Tac1* |
| 2 | rs30206506 | 6 | 7,512,626 | *Tac1* |
| 2 | rs30771076 | 6 | 7,512,775 | *Tac1* |
| 1 | rs3655269 | 6 | 17,922,618 | *-* |
| 1 | rs13478649 | 6 | 18,518,251 | *-* |
| 1 | rs13478656 | 6 | 21,893,927 | *-* |
| 1 | rs3684494 | 6 | 24,365,693 | *-* |
| 1 | rs13478697 | 6 | 32,800,650 | *Chchd3* |
| 1 | rs4139698 | 6 | 49,819,544 | *-* |
| 2 | rs13478762 | 6 | 54,175,671 | *Chn2* |
| 2 | rs30228387 | 6 | 54,224,143 | *Chn2* |
| 1 | rs3672029 | 6 | 75,345,665 | *-* |
| 1 | rs6285738 | 6 | 93,485,969 | *-* |
| 1 | rs6239023 | 6 | 94,005,991 | *Magi1* |
| 1 | rs6349084 | 6 | 96,697,598 | *-* |
| 1 | rs6339546 | 6 | 133,917,751 | *-* |
| 1 | rs13479053 | 6 | 134,201,252 | *Etv6* |
| 1 | rs3672808 | 6 | 139,805,730 | *Pik3c2g* |
| 1 | rs3711088 | 6 | 148,260,469 | *Tmtc1* |
| 1 | rs3659551 | 7 | 6,909,503 | *Usp29* |
| 1 | mCV23738426 | 7 | 8,465,811 | *Vmn2r52* |
| 1 | CEL-7_5627457 | 7 | 12,296,204 | *-* |
| 2 | rs32116079 | 7 | 19,147,099 | *Psg27* |
| 2 | rs13461382 | 7 | 19,591,025 | *Irf2bp1* |
| 2 | rs31505570 | 7 | 19,721,049 | *Fbxo46* |
| 1 | CEL-7_36725559 | 7 | 43,396,959 | *-* |
| 1 | rs4232449 | 7 | 48,581,740 | *-* |
| 2 | rs31525495 | 7 | 53,894,287 | *Sergef* |
| 2 | rs31708001 | 7 | 53,903,710 | *Tph1* |
| 2 | rs32373825 | 7 | 53,904,154 | *Tph1* |
| 2 | rs6279417 | 7 | 53,931,049 | *-* |
| 2 | rs6279463 | 7 | 53,931,082 | *-* |
| 2 | rs6281625 | 7 | 53,931,473 | *-* |
| 1 | rs6160140 | 7 | 73,426,174 | *Lrrk1* |
| 1 | rs3705155 | 7 | 75,667,606 | *-* |
| 1 | rs13479347 | 7 | 83,432,559 | *-* |
| 1 | rs13479355 | 7 | 85,431,231 | *Ntrk3* |
| 1 | rs13479358 | 7 | 86,843,104 | *5730590G19Rik* |
| 2 | rs31060727 | 7 | 107,753,415 | *Mrpl48* |
| 2 | rs32330100 | 7 | 107,755,420 | *Mrpl48 / Rab6* |
| 2 | rs32034601 | 7 | 107,756,141 | *Mrpl48 / Rab6* |
| 2 | rs31746209 | 7 | 107,758,176 | *Rab6* |
| 2 | rs31908266 | 7 | 107,771,055 | *Rab6* |
| 2 | rs32020539 | 7 | 107,791,025 | *Plekhb1* |
| 1 | rs3713052 | 7 | 108,918,190 | *Clpb* |
| 1 | rs6357312 | 7 | 109,389,815 | *Rhog* |
| 2 | rs13479460 | 7 | 118,818,343 | *Galntl4* |
| 1 | rs6194926 | 7 | 121,509,575 | *4933406I18Rik* |
| 1 | CEL-7_115892950 | 7 | 122,464,255 | *Rgs10* |
| 1 | CEL-7_122752866 | 7 | 129,495,978 | *Dock1* |
| 1 | rs13479506 | 7 | 131,822,778 | *3100003L05Rik* |
| 1 | rs3682038 | 7 | 133,483,665 | *-* |
| 1 | rs13479535 | 7 | 138,763,317 | *-* |
| 1 | rs3663988 | 7 | 146,505,067 | *-* |
| 1 | CEL-8_33812776 | 8 | 33,652,415 | *Tnks* |
| 2 | rs33319598 | 8 | 54,406,484 | *-* |
| 2 | rs32893761 | 8 | 54,441,823 | *-* |
| 2 | rs32900718 | 8 | 54,584,421 | *-* |
| 2 | rs37502172 | 8 | 54,664,031 | *-* |
| 1 | rs3707439 | 8 | 61,429,008 | *-* |
| 1 | rs13479807 | 8 | 68,141,750 | *Mar1* |
| 1 | rs13479811 | 8 | 69,577,205 | *-* |
| 1 | rs13479871 | 8 | 87,590,219 | *Fbxw9* |
| 1 | rs6257357 | 8 | 88,071,460 | *Dnaja2* |
| 1 | rs13479880 | 8 | 89,271,028 | *ENSMUSG00000074178* |
| 1 | rs13479884 | 8 | 90,183,245 | *-* |
| 1 | gnf08.108.032 | 8 | 106,104,093 | *Tmco7* |
| 1 | gnf08.118.027 | 8 | 116,136,203 | *-* |
| 1 | rs6400423 | 8 | 129,106,325 | *-* |
| 2 | rs3697596 | 8 | 130,354,209 | *-* |
| 1 | mCV25073238 | 9 | 10,596,883 | *-* |
| 1 | gnf09.012.310 | 9 | 17,874,218 | *-* |
| 1 | rs13480092 | 9 | 18,926,807 | *Olfr836* |
| 1 | rs3088801 | 9 | 24,802,456 | *-* |
| 1 | rs4135590 | 9 | 42,796,186 | *Arhgef12* |
| 1 | rs13480173 | 9 | 46,336,655 | *-* |
| 1 | rs3676124 | 9 | 83,013,939 | *Hmgn3* |
| 1 | rs3669564 | 9 | 87,780,386 | *-* |
| 1 | rs3711089 | 9 | 105,393,993 | *Atp2c1* |
| 1 | rs13480421 | 9 | 111,761,261 | *-* |
| 1 | rs6320810 | 9 | 115,065,092 | *Osbpl10* |
| 1 | rs3669563 | 9 | 117,827,882 | *-* |
| 1 | rs13459114 | 9 | 121,825,029 | *Cyp8b1* |
| 1 | rs13459119 | 10 | 20,045,489 | *Bclaf1* |
| 1 | rs3679120 | 10 | 22,641,085 | *-* |
| 1 | rs13480581 | 10 | 38,685,357 | *Lama4* |
| 2 | rs3090642 | 10 | 44,860,993 | *Prep* |
| 1 | rs13480630 | 10 | 67,283,841 | *-* |
| 1 | rs13480638 | 10 | 68,907,415 | *-* |
| 2 | rs29326309 | 10 | 95,590,622 | *-* |
| 2 | rs13480738 | 10 | 102,999,447 | *-* |
| 2 | rs13480739 | 10 | 103,185,336 | *-* |
| 1 | rs13480740 | 10 | 103,515,832 | *-* |
| 2 | rs6282517 | 10 | 103,725,176 | *-* |
| 1 | rs3688351 | 10 | 103,953,112 | *-* |
| 2 | rs13480742 | 10 | 103,966,226 | *-* |
| 2 | rs13480743 | 10 | 104,097,543 | *-* |
| 2 | rs13480744 | 10 | 104,441,816 | *100042383* |
| 2 | rs13480749 | 10 | 105,437,651 | *-* |
| 1 | rs6243755 | 10 | 108,174,849 | *Syt1* |
| 1 | rs13480773 | 10 | 114,179,165 | *Trhde* |
| 2 | rs6350239 | 10 | 114,513,832 | *Tph2* |
| 2 | rs4228474 | 10 | 114,515,997 | *Tph2* |
| 2 | rs29341895 | 10 | 114,624,510 | *-* |
| 2 | rs29354500 | 10 | 114,624,607 | *-* |
| 2 | rs29327697 | 10 | 114,625,405 | *-* |
| 1 | mCV24217147 | 10 | 117,503,347 | *Smgp21b* |
| 1 | rs13480803 | 10 | 122,568,425 | *Usp15* |
| 1 | mCV22832306 | 10 | 125,997,604 | *-* |
| 2 | rs3697243 | 10 | 126,250,067 | *-* |
| 1 | rs13480836 | 11 | 3,454,200 | *-* |
| 1 | rs6190775 | 11 | 6,312,209 | *-* |
| 1 | gnf11.017.294 | 11 | 18,007,065 | *-* |
| 1 | rs3723987 | 11 | 19,368,928 | *-* |
| 2 | rs13480933 | 11 | 29,118,539 | *Smek2* |
| 2 | rs26822202 | 11 | 29,122,316 | *Ccdc104* |
| 2 | rs29469152 | 11 | 29,122,634 | *Ccdc104* |
| 2 | rs29473241 | 11 | 29,147,356 | *Ccdc104* |
| 2 | rs26822189 | 11 | 29,147,449 | *Ccdc104* |
| 2 | rs29410558 | 11 | 29,149,274 | *-* |
| 1 | rs13459123 | 11 | 30,958,902 | *Asb3* |
| 2 | rs26950069 | 11 | 58,126,592 | *Zfp692* |
| 2 | rs29402173 | 11 | 58,128,820 | *Zfp672* |
| 2 | rs29406291 | 11 | 58,129,646 | *Zfp672* |
| 2 | rs29387701 | 11 | 58,138,073 | *1700047K16Rik* |
| 2 | rs29427167 | 11 | 58,138,512 | *-* |
| 2 | rs29397704 | 11 | 58,140,748 | *-* |
| 1 | rs3697686 | 11 | 58,381,052 | *Olfr327-ps1* |
| 1 | rs3711357 | 11 | 61,266,066 | *Zfp179* |
| 1 | rs13481061 | 11 | 62,806,119 | *Cdrt4* |
| 1 | rs13481071 | 11 | 65,014,320 | *Myocd* |
| 2 | rs26888740 | 11 | 74,739,049 | *Smg6 / Srr* |
| 2 | rs26888739 | 11 | 74,739,210 | *Smg6 / Srr* |
| 2 | rs26888734 | 11 | 74,740,289 | *Smg6* |
| 2 | rs6192434 | 11 | 74,745,116 | *Smg6* |
| 2 | rs29479272 | 11 | 74,978,579 | *Hic1* |
| 2 | rs6155957 | 11 | 74,978,877 | *Hic1* |
| 2 | rs28226774 | 11 | 76,806,816 | *-* |
| 2 | rs28226773 | 11 | 76,806,890 | *-* |
| 2 | rs28226748 | 11 | 76,840,125 | *Slc6a4* |
| 2 | rs28226747 | 11 | 76,840,970 | *Slc6a4* |
| 2 | rs28226743 | 11 | 76,841,856 | *Slc6a4* |
| 2 | rs28226734 | 11 | 76,846,429 | *-* |
| 1 | rs13481119 | 11 | 79,360,701 | *Nf1* |
| 1 | rs13481161 | 11 | 92,322,572 | *-* |
| 1 | rs13481313 | 12 | 14,876,064 | *-* |
| 1 | rs13481321 | 12 | 16,687,164 | *Greb1* |
| 1 | rs13481371 | 12 | 30,883,465 | *Sntg2* |
| 1 | rs6223000 | 12 | 34,867,610 | *-* |
| 1 | rs13481445 | 12 | 51,443,486 | *Prkd1* |
| 1 | rs3677344 | 12 | 65,698,044 | *-* |
| 1 | rs13481541 | 12 | 77,479,299 | *Zbtb1* |
| 1 | rs3662628 | 12 | 80,362,465 | *Zfyve26* |
| 1 | rs13481556 | 12 | 81,763,540 | *Slc39a9* |
| 1 | rs13481588 | 12 | 93,207,169 | *EG667589* |
| 1 | rs3023711 | 12 | 117,417,767 | *-* |
| 2 | rs3692361 | 12 | 118,957,587 | *Rapgef5* |
| 1 | rs13481673 | 13 | 5,584,894 | *-* |
| 1 | rs6348604 | 13 | 15,502,938 | *-* |
| 1 | rs13481706 | 13 | 16,432,650 | *-* |
| 1 | rs3710348 | 13 | 31,365,187 | *-* |
| 1 | rs3688207 | 13 | 45,454,657 | *-* |
| 2 | rs13462828 | 13 | 62,915,954 | *-* |
| 2 | rs30073403 | 13 | 63,213,965 | *2010111I01Rik* |
| 2 | rs29584318 | 13 | 63,383,551 | *2010111I01Rik* |
| 2 | rs29636134 | 13 | 63,405,500 | *Fancc* |
| 1 | rs13481871 | 13 | 71,432,597 | *-* |
| 1 | rs3686443 | 13 | 86,662,750 | *-* |
| 1 | rs3655061 | 13 | 89,723,142 | *Hapln1* |
| 1 | rs13481961 | 13 | 98,040,192 | *-* |
| 1 | rs4230144 | 14 | 8,924,022 | *Rpp14* |
| 1 | rs6322899 | 14 | 10,651,880 | *Fhit* |
| 1 | rs6290836 | 14 | 13,378,532 | *Cadps* |
| 2 | rs3696385 | 14 | 18,195,504 | *-* |
| 1 | rs13482104 | 14 | 27,163,606 | *-* |
| 1 | rs6396829 | 14 | 28,814,149 | *Erc2* |
| 1 | gnf14.055.608 | 14 | 55,370,255 | *Mipep* |
| 2 | rs3703075 | 14 | 63,543,436 | *Wdfy2* |
| 2 | rs13459144 | 14 | 76,317,480 | *Gtf2f2* |
| 1 | rs13482327 | 14 | 97,600,347 | *-* |
| 2 | rs3692362 | 14 | 100,175,288 | *-* |
| 1 | rs6191117 | 14 | 100,606,904 | *-* |
| 1 | rs3708779 | 14 | 111,167,655 | *EG668772* |
| 1 | rs13482375 | 14 | 112,079,790 | *Slitrk5* |
| 1 | rs6169105 | 14 | 117,657,346 | *Gpc6* |
| 1 | rs13459176 | 15 | 3,229,130 | *Sepp1* |
| 1 | CEL-15_9687257 | 15 | 9,601,488 | *-* |
| 1 | rs13482431 | 15 | 11,241,219 | *Adamts12* |
| 1 | rs3715857 | 15 | 19,181,733 | *-* |
| 1 | rs13482509 | 15 | 31,971,924 | *-* |
| 2 | rs32005588 | 15 | 32,841,248 | *-* |
| 2 | rs31983176 | 15 | 32,849,387 | *Sdc2* |
| 2 | rs32228111 | 15 | 32,858,088 | *Sdc2* |
| 2 | rs32250555 | 15 | 32,874,418 | *Sdc2* |
| 2 | rs4230687 | 15 | 32,964,635 | *Sdc2* |
| 2 | rs3695416 | 15 | 38,414,152 | *-* |
| 1 | rs3683326 | 15 | 41,219,409 | *-* |
| 1 | rs6400804 | 15 | 56,637,987 | *-* |
| 2 | rs36320059 | 15 | 81,147,953 | *Slc25a17* |
| 2 | rs37939180 | 15 | 81,148,568 | *Slc25a17* |
| 2 | rs37394767 | 15 | 81,148,663 | *Slc25a17* |
| 2 | rs38717462 | 15 | 81,151,100 | *Slc25a17* |
| 2 | rs36586819 | 15 | 81,167,126 | *Slc25a17* |
| 2 | rs36346494 | 15 | 81,192,154 | *-* |
| 2 | rs32320164 | 15 | 83,392,328 | *Tspo* |
| 2 | rs31717709 | 15 | 83,393,007 | *Tspo* |
| 2 | rs32046139 | 15 | 83,399,802 | *Tspo* |
| 2 | rs31565634 | 15 | 83,400,032 | *Tspo* |
| 2 | rs31717505 | 15 | 83,406,204 | *Ttll12* |
| 1 | rs13482712 | 15 | 92,076,446 | *Cntn1* |
| 1 | rs4152638 | 16 | 4,326,609 | *Adcy9* |
| 1 | rs4152790 | 16 | 4,850,158 | *4930562C15Rik* |
| 1 | rs4173902 | 16 | 37,352,904 | *Stxbp5l* |
| 1 | rs4177651 | 16 | 40,683,387 | *-* |
| 2 | rs3696661 | 16 | 51,676,815 | *-* |
| 1 | rs4197150 | 16 | 66,296,803 | *-* |
| 1 | rs3718160 | 16 | 76,867,900 | *-* |
| 1 | rs6317052 | 16 | 79,493,030 | *-* |
| 1 | rs4211364 | 16 | 80,830,317 | *-* |
| 1 | rs3672065 | 17 | 14,336,401 | *Dact2* |
| 1 | rs3726555 | 17 | 16,539,606 | *-* |
| 1 | rs13482899 | 17 | 17,761,307 | *Lnpep* |
| 1 | rs13482914 | 17 | 20,982,760 | *V1re8* |
| 2 | rs3696835 | 17 | 22,818,022 | *ENSMUSG00000046088* |
| 2 | rs3693494 | 17 | 29,917,069 | *-* |
| 2 | rs33798776 | 17 | 30,661,307 | *Btbd9* |
| 2 | rs33800126 | 17 | 30,695,419 | *-* |
| 2 | rs33798569 | 17 | 30,702,010 | *-* |
| 2 | rs33797503 | 17 | 30,703,669 | *-* |
| 2 | rs30778922 | 17 | 30,809,092 | *Dnahc8* |
| 2 | rs3145477 | 17 | 30,814,214 | *Dnahc8* |
| 1 | rs6298471 | 17 | 36,684,410 | *H2-M11* |
| 1 | rs6272475 | 17 | 53,622,040 | *Rab5a* |
| 1 | rs3714226 | 17 | 55,409,410 | *-* |
| 1 | rs3715723 | 17 | 58,810,428 | *-* |
| 1 | rs3675634 | 17 | 71,590,806 | *Lpin2* |
| 2 | rs33372832 | 17 | 74,733,598 | *-* |
| 2 | rs33595841 | 17 | 74,733,692 | *-* |
| 2 | rs33340320 | 17 | 74,733,758 | *-* |
| 2 | rs13465627 | 17 | 74,787,631 | *Spast* |
| 2 | rs33394982 | 17 | 74,790,564 | *-* |
| 1 | rs6229946 | 17 | 75,590,536 | *Ltbp1* |
| 1 | rs13483157 | 17 | 89,287,847 | *-* |
| 1 | rs6397044 | 17 | 93,594,172 | *-* |
| 1 | rs13483183 | 18 | 3,516,539 | *Bambi* |
| 1 | gnf18.001.688 | 18 | 4,647,835 | *-* |
| 2 | rs30263474 | 18 | 20,822,913 | *Ttr* |
| 2 | rs31313820 | 18 | 20,822,943 | *Ttr* |
| 2 | rs6273344 | 18 | 20,825,921 | *Ttr* |
| 2 | rs31315882 | 18 | 20,830,086 | *Ttr* |
| 1 | rs13483271 | 18 | 29,116,312 | *-* |
| 2 | rs29540760 | 18 | 31,595,494 | *Syt4* |
| 2 | rs29551386 | 18 | 31,598,369 | *Syt4* |
| 2 | rs30031611 | 18 | 31,600,912 | *Syt4* |
| 2 | rs30133602 | 18 | 31,683,612 | *EG383420* |
| 2 | rs29823717 | 18 | 31,699,317 | *-* |
| 2 | rs30303190 | 18 | 31,703,097 | *-* |
| 1 | rs3718586 | 18 | 33,291,841 | *Camk4* |
| 1 | rs3658163 | 18 | 68,820,968 | *-* |
| 1 | rs6161154 | 18 | 71,609,676 | *Dcc* |
| 1 | rs4137441 | 18 | 88,803,388 | *-* |
| 1 | rs13483525 | 19 | 10,518,533 | *Syt7* |
| 1 | rs6316813 | 19 | 11,396,714 | *Ms4a7* |
| 2 | rs38304960 | 19 | 12,859,265 | *Zfp91* |
| 2 | rs31193418 | 19 | 12,862,342 | *Zfp91* |
| 1 | rs6372656 | 19 | 21,917,692 | *Tmem2* |
| 1 | rs13483643 | 19 | 45,386,221 | *-* |
| 1 | rs6194426 | 19 | 50,203,520 | *-* |
| 1 | mCV23069572 | 19 | 52,369,475 | *-* |
| 1 | rs6304326 | 19 | 53,512,609 | *-* |
| 1 | rs6191324 | 19 | 59,396,320 | *Pdzd8* |
| 1 | gnfX.026.801 | X | 36,745,486 | *Stag2* |
| 1 | rs13483765 | X | 54,787,689 | *-* |
| 1 | gnfX.080.189 | X | 90,784,538 | *-* |
| 1 | rs13483894 | X | 93,694,393 | *Heph* |
| 1 | rs6182892 | X | 94,171,734 | *-* |
| 1 | rs6221690 | X | 127,572,284 | *-* |
| 1 | rs13483997 | X | 128,648,050 | *-* |
| 1 | rs13484004 | X | 130,310,074 | *-* |
| 2 | rs3697198 | X | 132,751,198 | *Tceal7* |
| 1 | rs13484043 | X | 139,242,663 | *Tmem164* |
| 1 | gnfX.148.995 | X | 162,427,214 | *Arhgap6* |
