# Supplementary figures and images for: The endocrine stress response is linked to one specific locus on chromosome 3 in a mouse model based on extremes in trait anxiety
Source: BMC Genomics. 2012 Oct 31;13:579. doi: 10.1186/1471-2164-13-579 (PMC3557225; doi:10.1186/1471-2164-13-579)

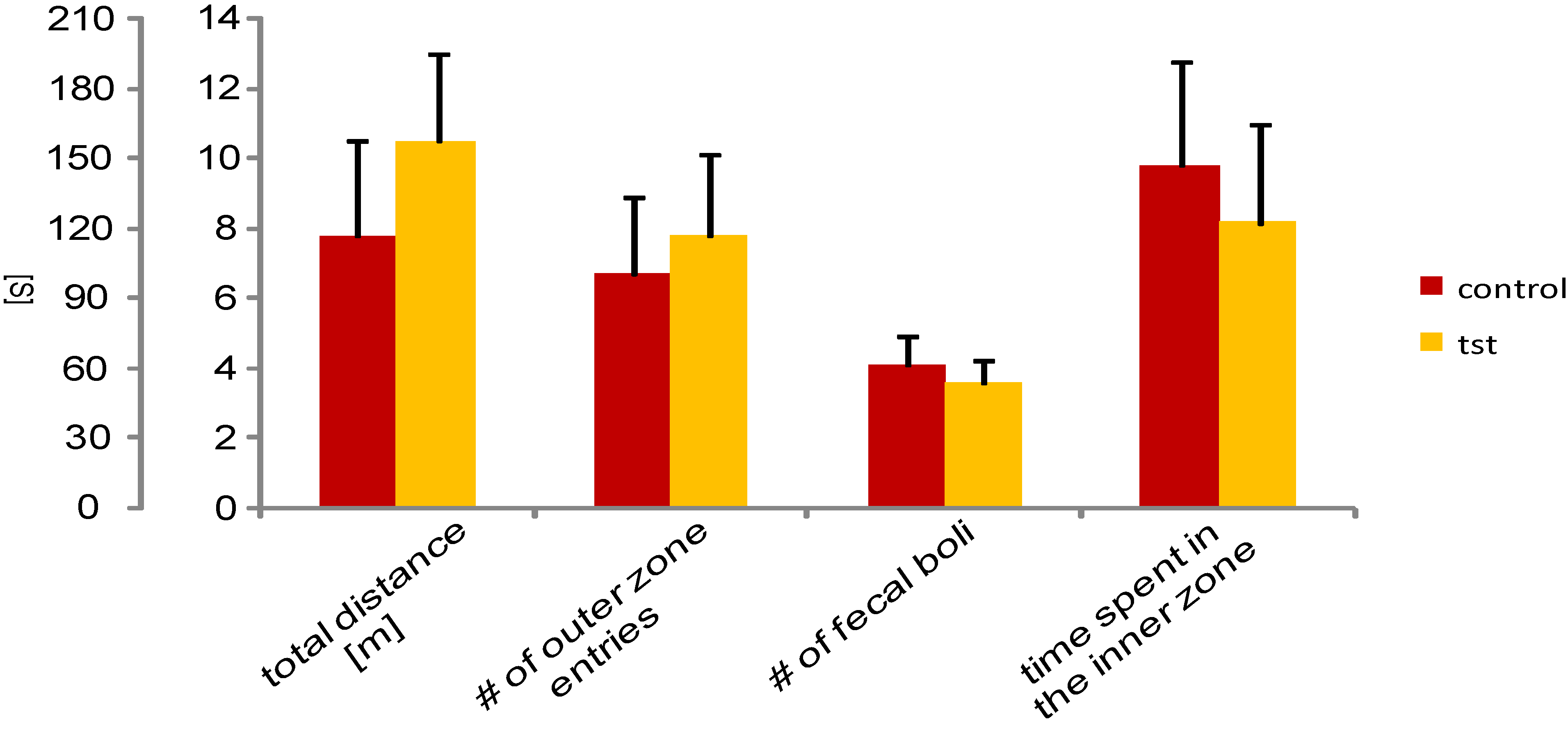

Supplement: Additional file 2 — Figure S1. Effect of prior tail-suspension test exposure on the behaviour in the open field test. Key parameters assessed in the open field test showed no significant difference in CD-1 mice between control (N = 10, only exposed to the open field test) and tst (N = 9, exposed to the tail-suspension test 48 h before the open field test) mice. Data are shown as means +SEM. [file 1471-2164-13-579-S2.tiff]
